# The Effects of NMDA Receptor Blockade on Sensory-Evoked Responses in Superficial Layers of the Rat Barrel Cortex

**DOI:** 10.3389/fncel.2019.00259

**Published:** 2019-06-07

**Authors:** Julia Lebedeva, Andrey Zakharov, Gulshat Burkhanova, Kseniya Chernova, Roustem Khazipov

**Affiliations:** ^1^Laboratory of Neurobiology, Kazan Federal University, Kazan, Russia; ^2^Department of Physiology, Kazan State Medical University, Kazan, Russia; ^3^Aix Marseille University, INSERM, INMED, Marseille, France

**Keywords:** NMDA receptor, barrel cortex, epipial application, APV, sensory-evoked potential

## Abstract

Transmission of excitation from L4 to L2/3 is a part of a canonical circuit of cortical sensory signal processing. While synapses from L4 to L2/3 are mediated by both AMPA and NMDA glutamate receptors, previous studies suggested that sensory-evoked excitation of neurons in supragranular layers is almost entirely mediated by NMDA receptors. Here, we readdressed this question using extracellular recordings of sensory-evoked potentials (SEPs) and multiple unit activity (MUA) in the rat barrel cortex. We found that blockade of NMDA receptors using the selective antagonist dAPV profoundly inhibited the late part of L2/3 SEP, the associated sink, and MUA response but did not affect its initial part. Our results indicate that both non-NMDA and NMDA receptors are involved in sensory signal transmission from L4 to L2/3. While non-NMDA receptors mediate fast transmission of sensory signals, NMDA-Rs are importantly involved in the generation of the late phase of the sensory-evoked response in supragranular layers.

## Introduction

The canonical circuit of signal processing in the sensory cortex involves thalamus driven activation of L4 neurons followed by a transfer of excitation from L4 to L2/3 (Gilbert and Wiesel, 1979; Petersen, 2007; Feldmeyer et al., 2013). In the barrel cortex, excitation of L2/3 neurons during sensory-evoked responses is characterized by sparse firing within a time window of ∼ 100 ms after whisker deflection (Simons, 1978; Armstrong-James et al., 1993; Brecht et al., 2003; Kerr et al., 2007; de Kock et al., 2007; Constantinople and Bruno, 2011; Reyes-Puerta et al., 2015). Experimental data and the results of modeling studies indicate that neuronal excitation in L2/3 during sensory response is brought by summation of excitatory postsynaptic potentials (EPSPs) at L4 - L2/3 synapses and recurrent synapses in the L2/3 network (Brecht et al., 2003; Sarid et al., 2007, 2015; de Kock et al., 2007; Lefort et al., 2009). Both of these types of connections involve activation of AMPA and NMDA receptors, which mediate the fast and slow components of EPSPs in L2/3 neurons, respectively (Feldmeyer et al., 2002, 2006; Busetto et al., 2008). In keeping with this, pharmacological suppression of cortical AMPA and NMDA receptors completely suppresses L2/3 sensory-evoked potentials (SEPs) and multiple unit responses evoked by whisker stimulation (Vinokurova et al., 2018). However, the relative contribution of these two types of glutamate receptors to the SEPs and excitation of L2/3 neurons remains incompletely understood.

Recording single unit activation in response to tactile stimuli in the barrel cortex revealed that suppression of cortical NMDA-Rs almost completely suppressed all spikes from L2/3 (Armstrong-James et al., 1993). These latter findings suggested that NMDA-Rs primarily mediate signal flow from L4 to L2/3, and that despite the well-established contribution of AMPA receptors to synaptic transmission to L2/3 neurons, AMPA-Rs have little contribution to the interlayer signal transfer during sensory-evoked responses. However, because single unit recordings may provide incomplete information on population dynamics, in the present study we readdressed the effects of cortical NMDA-R suppression on sensory-evoked responses in the rat barrel cortex using extracellular recordings of SEPs and multiple unit activity (MUA). We report that both AMPA and NMDA receptors contribute to the excitation transfer from L4 to L2/3 and mediate the early and late parts of the sensory-evoked response, respectively.

## Materials and Methods

### Ethical Approval

This work has been carried out in accordance with EU Directive 2010/63/EU for animal experiments, and all animal-use protocols were approved by the French National Institute of Health and Medical Research (INSERM, protocol N007.08.01) and Kazan Federal University on the use of laboratory animals (ethical approval by the Institutional Animal Care and Use Committee of Kazan State Medical University N9-2013).

### Surgery

Wistar rats of either sex from postnatal day P19-27 (P0 = day of birth) were used. Surgery was performed under isoflurane anesthesia (4% for induction, 2% for maintenance), and urethane (1 g/kg, i.p.) was injected at the end of surgery. The skull of the animal was cleaned of skin and periosteum, dried and covered with cyanacrylamide glue except for a 4–9 mm^2^ window above the left barrel cortex. Then the skull was covered with dental cement (Grip Cement, Caulk Dentsply, DE, United States). A metal ring (15 mm inner diameter) was fixed to the rat’s head by dental cement. The area inside the ring was left cement-free. After surgery, the animals were warmed, and left for 1 h to recover. The head was attached to a ball-joint holder by the metal ring. During recordings animals were heated via a thermal pad (35–36°C). A chlorided silver wire, placed in the visual cortex, served as the ground electrode.

A cranial window ∼3–4 mm in diameter was drilled above the barrel cortex (window edges: AP -0.5 to -4.5 mm; lateral 2.5–6.5 mm from bregma) (Paxinos and Watson, 2007; Khazipov et al., 2015), and a 1–2 mm-long dura incision was made in the middle of the cranial window using a 27G needle. Dura dissection was carried out carefully to avoid bleeding and the formation of blood clots on the brain surface, which could obstruct drug diffusion. The cranial window was encircled with a 1 mm-high cement wall to form an epipial chamber. Artificial cerebrospinal fluid (ACSF) during control recordings and dAPV dissolved in ACSF was applied to the epipial chamber at 20–40 μl volumes every 5–10 min as described previously (Vinokurova et al., 2018).

### Extracellular Recordings

Extracellular local field potentials (LFP) and MUA were recorded from a single barrel column using 16-site linear silicon probes (100 μm separation distance between recording sites, Neuronexus Technologies, MI, United States). Probes were inserted perpendicularly to the cortical surface to a depth of 1300–1900 μm (depending on animal age). The signals from extracellular recordings were amplified and filtered (x10,000; 0.15 Hz–10 kHz) using a DigitalLynx (Neuralynx, United States) amplifier, digitized at 32 kHz and saved on a PC for *post hoc* analysis. The whiskers were trimmed to a length of 0.8–1.5 mm and were stimulated using a piezoelectric bending actuator (Noliac, Denmark) using 200 ms square pulses with 5–10 s intervals. A needle (22G) was glued to the end of a piezo actuator and the tip of the whisker was inserted into the blunt tip of the needle. The principal whisker (PW) was identified by the shortest latency MUA responses in layer 4 evoked by single whisker deflection.

### Data Analysis and Statistics

Raw data were processed using MATLAB environment (MathWorks, United States). The wide-band signal was downsampled to 1000 Hz by the mean function *resample* and used as the LFP signal. Positive polarity is shown as up in all figures. For action potential detection, the raw wide-band signal was filtered (bandpass 300–5000 Hz) and negative deflections exceeding 5 standard deviations calculated over the most silent 1 s segment of the filtered trace were considered as spikes (multiple unit activity, MUA). LFPs and MUA were analyzed by custom-developed, MATLAB-based programs including ExpressAnalysis and Eview (AZ^1^). Sensory evoked potentials (SEPs) were detected as the first LFP troughs of sensory evoked ON-responses. SEP onset was detected at the intersection of the SEP front with the baseline. SEP peak amplitude corresponded to the maximal value of the first LFP trough, and its latency corresponded to a delay of SEP peak from the stimulus onset. SEP slope was calculated as an extreme value of the SEP’s front first derivative. Current source density (CSD) was computed as second spatial derivative of LFP for each recording site excluding the top and bottom sites with linear interpolation and plotted as pseudocolor images. CSD was calculated after subtraction of baseline (1 s long segment of average LFP before stimulus).

The cortical depth of the individual recording sites was estimated stereotaxically. The earliest sinks near an expected depth 600 μm along with short latency spikes served to identify L4, with 2 channels selected for MUA analysis. Data for L2/3 were sampled from a cortical depth >100 μm below the cortical surface and >100 μm above L4. With a separation distance of 100 μm between channels, two to three recording sites (on average, 2.8 ± 0.4 channels) were considered for sampling MUA from L2/3. L2/3 LFP and CSD signals were analyzed from the middle site in L2/3 located at a depth of 360 ± 25 μm from the cortical surface.

Analysis of MUA dynamics during the early part of the sensory-evoked response was performed by calculation of continuous MUA frequency using a 1 ms sliding window with 0.2 ms steps. Onset, the first peak density and its delay, and the rising slope of MUA were detected using algorithms similar to those described above for SEP analysis. MUA analysis through the entire time course of the response was calculated as mean firing rate corrected for baseline activity within five post stimulus time epochs (5–8, 8–10, 10–20, 20–50, and 50–100 ms) introduced by Armstrong-James et al. (1993). The mean relative MUA decrement with dAPV within epochs was calculated by normalizing the mean difference of MUA density in control and in the presence of dAPV to the control values [(control – dAPV)/control].

Statistical analysis was performed using the Matlab Statistics toolbox. Group data are presented as mean ± standard error. Shaded areas on figures also indicate standard error. The two-sided signed rank test for the matched samples was performed to assess the significance of differences between groups of data with the level of significance kept at *p* < 0.05.

### Drugs

Urethane and dAPV (D(-)-2-Amino-5-phosphonopentanoic acid) were purchased from Sigma. Isoflurane was purchased from Baxter. The concentration of dAPV (2 mM) for epipial application was selected on the basis of the results of a previous study (Vinokurova et al., 2018).

## Results

In the present study we explored the effects of blockade of cortical NMDA receptors using epipial application of the selective NMDA receptor antagonist dAPV on PW – evoked responses in L2/3 of a cortical barrel column. In total, 10 animals were used in this study and all values are presented below as a mean ± standard error. dAPV (2 mM) was applied epipially at the site of electrode insertion into the cortex and its effects on the sensory-evoked response were analyzed during the time period from 40 to 50 min after the onset of dAPV application, when the effects of the drug attained steady-state and presumably saturating levels in keeping with the results of previous studies (Vinokurova et al., 2018).

**FIGURE 1:**
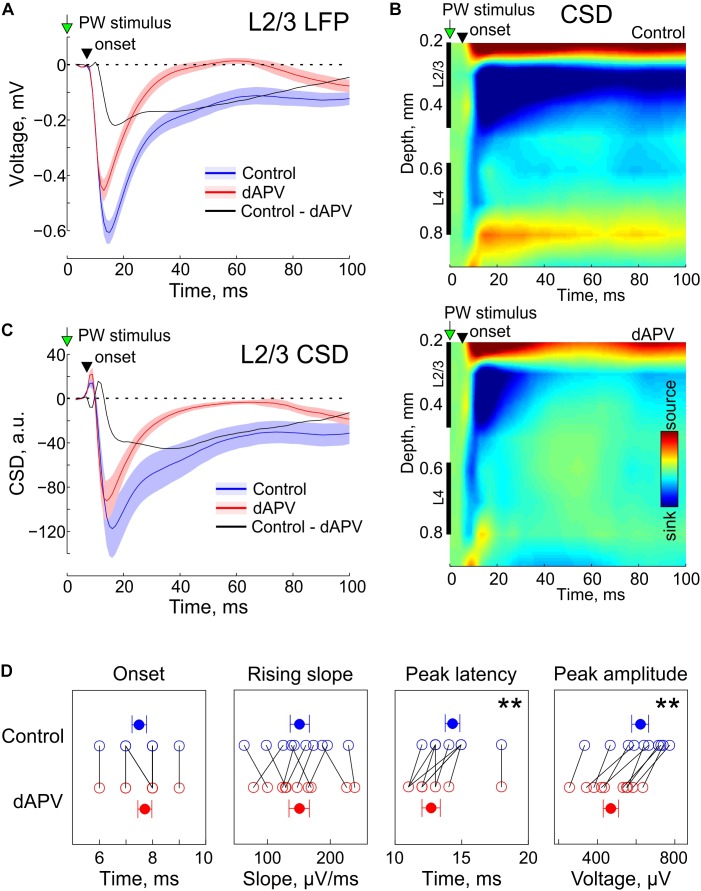
Effect of epipial dAPV on sensory-evoked potential (SEP) in L2/3 of the rat barrel cortex.**(A)** Grand average L2/3 SEP evoked by the PW deflection in control (blue) and 40 to 50 min of 2 mM – dAPV application (red). Shaded areas show SE. Time = 0 corresponds to the onset of the PW deflection. Pooled data from 10 rats. The black trace shows the difference between the responses in control conditions and in the presence of dAPV. **(B)** Current-source density (CSD) map of an average sensory-evoked response in control conditions (top) and in the presence of dAPV (bottom). **(C)** Grand average CSD in the middle of L2/3 corresponding to the responses shown on panel **A**. **(D)** Group data on the effects of epipial dAPV on L2/3 SEP onset latency, peak latency, peak amplitude and maximal slope. Each open circle corresponds to an individual animal, filled circles show group average ± SE. The significance value of *p* was obtained through a two-sided signed rank test for the matched samples (^∗∗^*p* < 0.01).

In control conditions, brief deflections of the PW evoked characteristic L2/3 response consisting of a SEP (Figure 1A) associated with a sink on the CSD map (Figure 1B,C). As shown on the grand average responses on Figure 1A, SEP onset was detected 7.5 ± 0.3 ms after the stimulus onset and its rising slope was 151 ± 15 μV/ms. dAPV affected none of the parameters of the early part of the response (SEP onset: 7.7 ± 0.3 ms, *p* = 0.50, two-sided signed rank test for the matched samples; SEP rising slope: 151 ± 16 μV/ms; *p* = 0.92). However, dAPV reduced the peak SEP amplitude from 621 ± 43 μV to 469 ± 38 μV (*p* = 0.002) and reduced the SEP peak delay after the stimulus onset from 14.3 ± 0.5 ms to 12.7 ± 0.7 ms (*p* = 0.008). dAPV also profoundly inhibited the late part of the SEP with the maximal level of inhibition observed within a time window from 20 to 60 ms after the stimulus. When measured at the time point of 50 ms after the stimulus, negative LFP deflection was reduced in the presence of dAPV from −147 ± 31 μV to 1 ± 15 μV (*p* = 0.002). These effects of dAPV on SEP parameters were similar among all animals in the group (Figure 1D).

We further decomposed the dAPV-sensitive, NMDA receptor dependent component of SEP by subtracting the d-APV resistant SEP component from the control response. These two pharmacologically isolated SEP components, the dAPV-insensitive non-NMDA receptor dependent component (Figure 1A, red trace), and the dAPV – sensitive NMDA receptor dependent SEP component (Figure 1A, black trace) contributed differently to the L2/3 SEP through the time course of the sensory response. The initial part of the response was mainly mediated by the dAPV – insensitive component, whereas its late part was predominantly NMDA receptor dependent. At 10.5 ± 0.3 ms after the stimulus (3 ms after the SEP onset), the dAPV – insensitive component accounted for 96 ± 6% of the SEP whereas the contribution of the dAPV- sensitive component was minimal (4 ± 6%). At its peak (12.6 ± 0.6 ms), the dAPV- insensitive component contributed 83 ± 4% to the negative LFP signal whereas the contribution of the dAPV – sensitive component was 17 ± 4%. At the peak of the control response which occurred at 14.3 ± 0.5 ms, the relative contribution of the dAPV-insensitive and dAPV-sensitive components were 69 ± 4% and 31 ± 4%, respectively. At the first peak of the d-APV sensitive component (17.5 ± 0.6 ms) the relative contributions of these two components were 58 ± 4% and 42 ± 4%. Through the late parts of the response, the relative contribution of the dAPV-insensitive component progressively decreased. At the second peak of the dAPV-insensitive component (46 ± 5 ms), the SEP was almost entirely mediated by the dAPV-sensitive component (88 ± 12%). CSD analysis of SEPs revealed a similar contribution of these two dAPV-sensitive and dAPV-insensitive components to the L2/3 sinks associated with SEP (Figure 1B,C).

**FIGURE 2:**
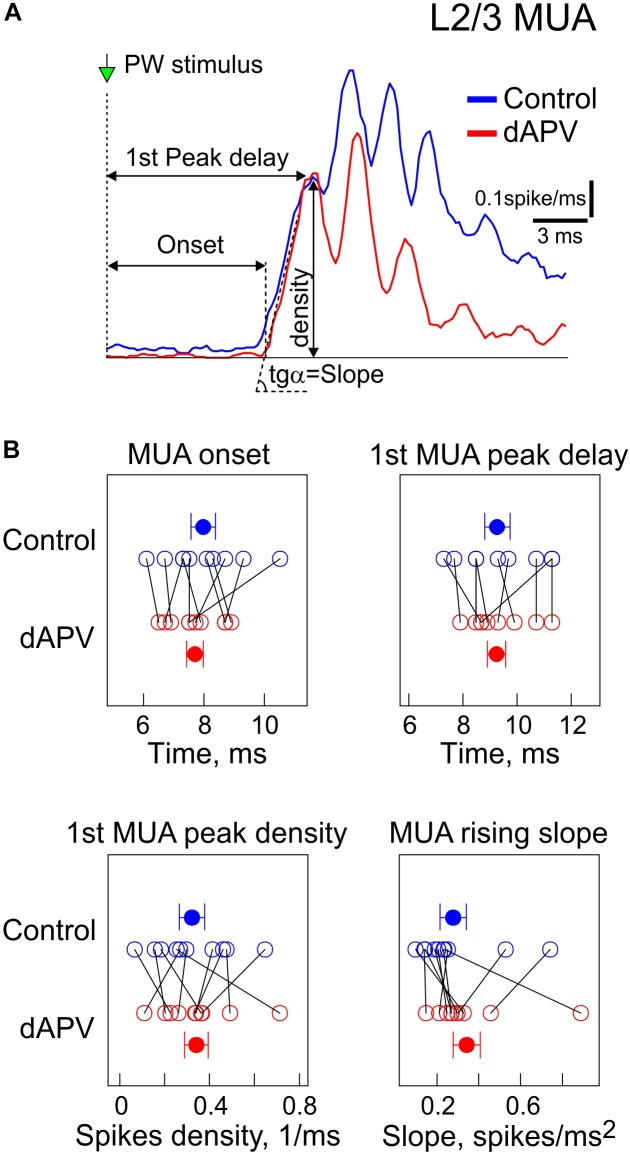
Effect of dAPV on the initial part of the sensory-evoked MUA. **(A)** Example average spike frequency before (blue) and after (red) application of dAPV in L2/3 (200–400 μm depth) with indication of how parameters of the MUA onset latency, the first MUA peak latency and density, and MUA slope at the onset of response and peak amplitude were calculated. **(B)** Group data on effects of epipial dAPV on the L2/3 MUA onset latency, latency and density of the first MUA and slope peak amplitude and MUA slope. Each open circle corresponds to an individual animal, filled circles show group average ± SE. Significance value of *p* was obtained through a two-sided signed rank test for matched samples.

**FIGURE 3:**
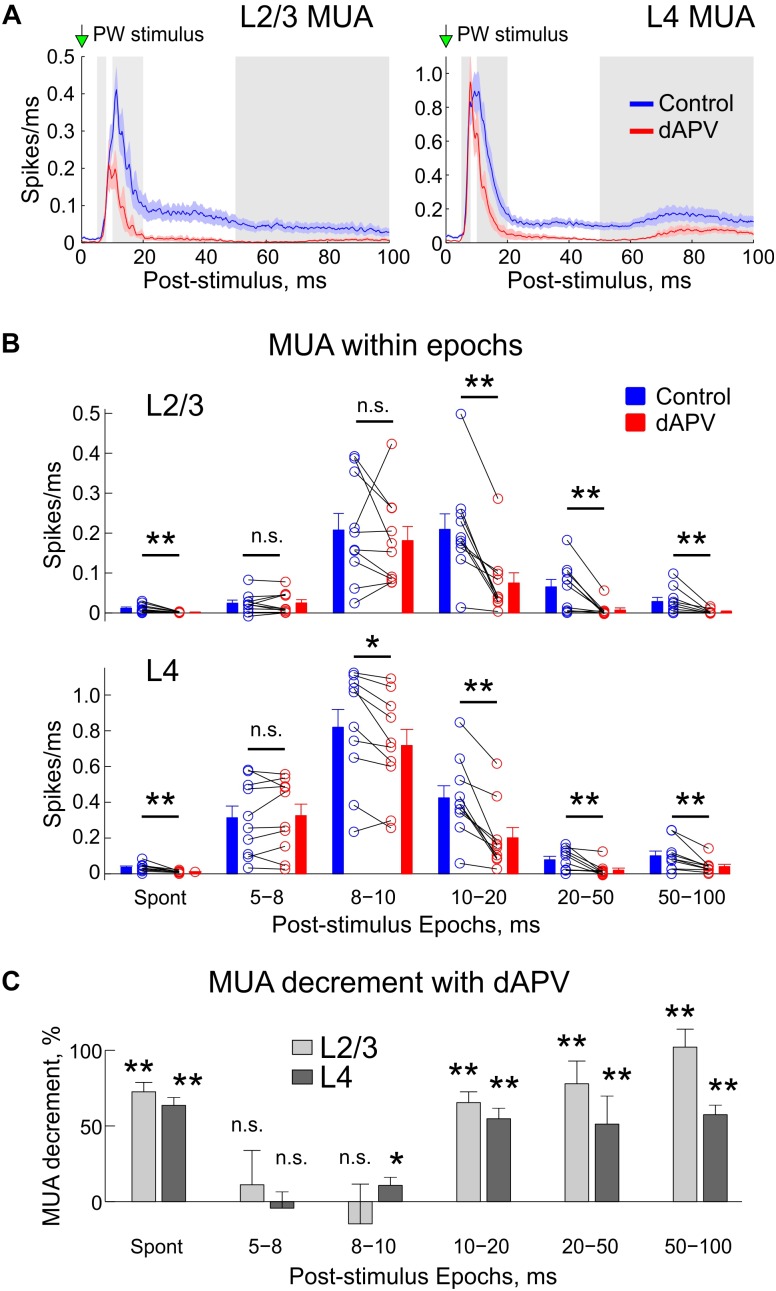
Inhibitory effect of the epipial dAPV on MUA through the entire time course of the sensory-evoked response. **(A)** Average sensory-evoked L2/3 (left) and L4 (right) MUA response in control conditions (blue) and 40 to 50 min after application of 2 mM – dAPV (red). Alternating white and gray zones indicate the epochs used in the analysis shown on panels **B,C**. **(B)** Group data on the effects of epipial dAPV on MUA density in different epochs of the sensory-evoked MUA response in L2/3 (top) and L4 (bottom). Epoch division was adapted from Armstrong-James et al. (1993) for comparisons. Spont, spontaneous MUA. Each open circle corresponds to an individual animal, columns and error bars show group average ± SE. Significance value of *p* was obtained through a two-sided signed rank test for the matched samples (^∗^*p* < 0.05, ^∗∗^*p* < 0.01). **(C)** Level of the dAPV-induced L2/3 (light gray) and L4 (dark gray) MUA suppression (“MUA decrement”) within the Armstrong-James’ epochs, calculated from the data shown on panel **B** as (control – dAPV)/control MUA.

We further analyzed the effects of dAPV on MUA through the time course of the sensory-evoked response. Several parameters of the sensory-evoked L2/3 MUA response were analyzed as shown on Figure 2A. The onset of the sensory-evoked L2/3 MUA responses was observed 7.9 ± 0.4 ms after the stimulus onset, and this was unchanged in the presence of dAPV (7.7 ± 0.3 ms, *p* = 0.79) (Figure 2A,B). The early phase of L2/3 MUA response was characterized by several peaks occurring at 398 ± 4 Hz which corresponds to high-frequency oscillations (Kandel and Buzsaki, 1997; Jones and Barth, 1999; Jones et al., 2000; Barth, 2003; Vinokurova et al., 2018). The first L2/3 MUA peak of 0.32 ± 0.05 spikes/ms was attained 9.3 ± 0.5 ms after the stimulus onset, with a rising slope of 0.28 ± 0.06 spikes/ms^2^. None of the following parameters which characterize the early part of the response were affected by dAPV: first MUA peak amplitude: 0.34 ± 0.5 spikes/ms (*p* = 1), delay of the first MUA peak: 9.2 ± 0.3 ms (*p* = 0.75), the rising slope of the first peak: 0.34 ± 0.07 spikes/ms^2^ (*p* = 0.49). However, the later L2/3 MUA response was inhibited by dAPV. The maximal peak of the L2/3 MUA response which attained a value of 0.56 ± 0.06 spikes/ms 11.5 ± 0.4 ms after the stimulus was reduced in the presence of dAPV to 0.37 ± 0.05 spikes/ms (see also Figure 3A).

We further quantified the effects of dAPV on L2/3 and L4 MUA during different time epochs of the sensory-evoked response according to Armstrong-James et al. (1993), which are shown as alternating white and gray areas on Figure 3A. Absolute values of MUA frequency during each epoch in each individual animal in control conditions and after application of dAPV are shown on Figure 3B. The mean relative MUA decrement with dAPV within epochs calculated by normalizing the mean difference of MUA density in control and in the presence of dAPV to the control values ((control – dAPV)/control) is shown on Figure 3C. In keeping with the results of analysis above, in L2/3 we failed to observe any significant change within the first 5–8 ms and 8–10 ms epochs after the stimulus. In L4, the MUA response was also non-affected during the first 5–8 ms epoch, and was reduced by only 11 ± 5% (*p* = 0.014) during the 8–10 ms epoch. During the later epochs, the MUA response was strongly inhibited: during the 10–20 ms epoch by 65 ± 7% (*p* = 0.002) in L2/3 and by 55 ± 7% (*p* = 0.002) in L4, during the 20–50 ms epoch by 78 ± 7% (*p* = 0.004) in L2/3 and by 51 ± 19% (*p* = 0.002) in L4, and during the 50–100 ms epoch by 102 ± 7% (*p* = 0.002) in L2/3 and by 57 ± 7% (*p* = 0.004) in L4.

## Discussion

In the present study, we explored the effects of the selective NMDA-R antagonist dAPV on the SEPs and MUA in L2/3 of cortical barrel columns. We found that dAPV did not affect the initial part of the sensory-evoked responses including the initial SEP slope, sink and short-latency MUA. However, dAPV reduced SEP amplitude, shortened SEP peak delay and profoundly inhibited the late part of SEP and MUA. Our results suggest that fast transmission of excitation from L4 to L2/3 primarily involves activation of AMPA receptors whereas NMDA-Rs are mainly involved in the activation of L2/3 neurons during the late part of the sensory-evoked response.

Present results are in general agreement with current models of signal transfer from L4 to L2/3, which involve activation of AMPA and NMDA receptors at L4 → L2/3 and L2/3 → L2/3 excitatory synapses during sensory responses (Laaris et al., 2000; Brecht et al., 2003; Petersen et al., 2003; de Kock et al., 2007; Sarid et al., 2007, 2015). We found that the initial part of SEP and MUA responses are unchanged by dAPV, and that the d-APV resistant component of the sensory-evoked response is characterized by fast rise kinetics and decay to the baseline within 30 ms (Figure 1). We have previously shown that under similar experimental conditions, these early and late parts of sensory-evoked responses are fully suppressed in L2/3 by combined application of dAPV and the AMPA receptor antagonist CNQX indicating the dAPV-resistant component is primarily mediated by AMPA receptors (Vinokurova et al., 2018). However, the rise-time (20–80% of 2.0 ± 0.2 ms) and decay time constant (11.1 ± 1.6 ms) of this dAPV resistant LFP response were significantly slower than the kinetics of the unitary AMPA receptor mediated monosynaptic EPSCs at the L4 - L2/3 synapses with their rise time of 0.33 ms and decay time constant of 1.86 ms (Feldmeyer et al., 2002). This suggests that the dAPV resistant component of SEP involves temporal summation of the AMPA receptor mediated desynchronized inputs from L4 and recurrent synapses in L2/3.

Interestingly, this dAPV-resistant MUA response was not seen in a previous study (Armstrong-James et al., 1993) in which the effects of dAPV on sensory-evoked responses were assessed using single cell recordings. Also, L2/3 cells in that study as well as in other studies using patch-clamp recordings barely fired action potentials with a short <10 ms latency under control conditions (Armstrong-James et al., 1993; Brecht et al., 2003; de Kock et al., 2007; Constantinople and Bruno, 2013). Although the reasons for this discrepancy are unknown, the difference in the recording techniques, sampling of single cells in the above cited studies versus MUA recordings in the present study, which provides a description of average activity in a larger population, could be an explanation. In our recordings, on average 24 ± 5 MUA spikes per response were detected during the 200 ms time window after the stimulus from 2 to 3 electrodes (on average, 2.8 ± 0.4 electrodes) located in L2/3. Assuming that L2/3 cells fire at 0.2 probability during sensory-evoked response (Kerr et al., 2007) we estimated that action potentials from 118 ± 28 L2/3 neurons were sampled in our MUA recordings from each animal. Also, given that firing in a short latency (<10 ms after stimulus) window accounted for only a small (11 ± 1%) fraction of all spikes during responses, and in keeping with the low probability of firing, the probability of detecting sensory-evoked spikes at short latency during single-cell recordings should also be low. In addition to this “probabilistic” explanation, one can not exclude the participation of spikes from the axon terminals of (i) L4 cells to L2/3 MUA at the response onset as has been described for the short latency spikes representing a thalamic afferent volley in L4 (Swadlow and Gusev, 2002; Swadlow et al., 2002; Hagen et al., 2017) and (ii) a thalamic afferent volley in L3 (Jensen and Killackey, 1987; Arnold et al., 2001).

The late part of the sensory-evoked response was profoundly inhibited by dAPV, including suppression of the late SEP and MUA. Decomposition of the NMDA receptor dependent component of the sensory-evoked response through subtraction of the dAPV-resistant component from the control response revealed that its time course is compatible with the slow time course of the NMDA receptor mediated postsynaptic currents. The dAPV sensitive SEP component was characterized by a rise-time (20–80%) of 3.9 ± 0.9 ms, a time to peak from the stimulus of 24 ± 3 ms (∼16 ms from the response onset) and biphasic decay with the second peak at 46 ± 5 ms after the stimulus, which was also evident in L2/3 sinks during CSD analysis and which likely has a “polysynaptic” nature (Crepel et al., 1997). Exponential fit of the dAPV sensitive SEP component provided the decay time constant of 88.3 ± 23.8 ms. Both the rise time and the decay of this SEP component were significantly slower than that of the unitary NMDA receptor mediated component of EPSCs at L4-L2/3 synapses (20–80% rise time of 2.1 ms and the decay time constant of 26 ms (Feldmeyer et al., 2002)). Moreover, the decay of the dAPV-sensitive SEP component and associated sink displayed a second peak which is different from the monoexponential decay of monosynaptic NMDA receptor mediated EPSCs (Feldmeyer et al., 2002).

We suggest that the dAPV-sensitive SEP component is complex and can be divided into two parts. The initial part of the dAPV-sensitive SEP component likely involves temporal summation of NMDA receptor mediated currents at L4 to L2/3 synapses which are activated during the initial part of the response. The late part of the dAPV-sensitive SEP component likely has a “polysynaptic” nature involving activity of L4 and L2/3 units during late response epochs (both profoundly suppressed by dAPV) and summation of both AMPA and NMDA receptor mediated currents at L4 to L2/3 synapses and recurrent L2/3 synapses. The particular sensitivity of the late part of response to dAPV indicates critical involvement of NMDA receptors, whose activation likely involves relatively low Mg^2+^ sensitivity and longer kinetics of NMDA-R mediated postsynaptic currents in excitatory neurons due to the presence of the NR2C subunit in cortical excitatory neurons (Monyer et al., 1994; Binshtok et al., 2006). This is in keeping with prominent NMDA-R mediated EPSPs evoked at resting membrane potential and physiological Mg^2+^ concentrations in paired recordings between L4 and L2/3 neurons (Feldmeyer et al., 2002). Secondly, the late part of sensory-evoked response involves temporal summation of postsynaptic currents, which is more efficient in the case of NMDA receptors with their longer kinetics as it occurs, for example, during generation of delta-waves associated with spindle-bursts in the immature somatosensory cortex (Minlebaev et al., 2009), and during the disinhibition-induced polysynaptic response in L2/3 of the neocortex (Laaris et al., 2000) and CA1 in the hippocampus (Crepel et al., 1997).

## Conclusion

In conclusion, our results suggest that both AMPA and NMDA receptors are involved in the generation of SEP and excitation of L2/3 neurons during sensory-evoked responses. While the initial part of SEP and fast transmission of excitation from L4 to L2/3 during sensory response primarily involves activation of AMPA receptors, NMDA-Rs are critically involved in the generation of the late part of SEP and delayed activation of L2/3 neurons. In future studies, it would be of interest to verify these hypotheses in modeling experiments by taking into account the participation of inhibitory conductances and multiple dipoles contributing to SEP generation.

## Data Availability

Original and processed data, and signal processing and analysis routines including the Eview and ExpressAnalysis software are available on request from the authors.

## Ethics Statement

This work has been carried out in accordance with EU Directive 2010/63/EU for animal experiments, and all animal-use protocols were approved by the French National Institute of Health and Medical Research (INSERM, protocol N007.08.01) and Kazan Federal University on the use of laboratory animals (ethical approval by the Institutional Animal Care and Use Committee of Kazan State Medical University N9-2013).

## Author Contributions

RK conceived the project and wrote the manuscript. JL, GB, and KC performed the experiments. AZ analyzed the data.

## Conflict of Interest Statement

The authors declare that the research was conducted in the absence of any commercial or financial relationships that could be construed as a potential conflict of interest.

## Footnotes

1 https://github.com/AndreyZakharovExp
